# Understanding and valuing the broader health system benefits of Uganda’s national Human Resources for Health Information System investment

**DOI:** 10.1186/s12960-015-0036-0

**Published:** 2015-08-31

**Authors:** Julia Driessen, Dykki Settle, David Potenziani, Kate Tulenko, Twaha Kabocho, Ismail Wadembere

**Affiliations:** University of Pittsburgh, Crabtree A614, 130 De Soto St., Pittsburgh, PA 15261 USA; IntraHealth International, 6340 Quadrangle Drive, Suite 200, Chapel Hill, NC 27517 USA; CapacityPlus, IntraHealth International, 1776 Eye Street, NW, Washington, DC 20006 USA; Pwani Health Informatics, Pwani University, Kilifi, Kenya; IntraHealth International, Kampala, Uganda

**Keywords:** Human resources information system, HRIS, Human resources for health, Global health, Workforce strengthening

## Abstract

**Background:**

To address the need for timely and comprehensive human resources for health (HRH) information, governments and organizations have been actively investing in electronic health information interventions, including in low-resource settings. The economics of human resources information systems (HRISs) in low-resource settings are not well understood, however, and warrant investigation and validation.

**Case description:**

This case study describes Uganda’s Human Resources for Health Information System (HRHIS), implemented with support from the US Agency for International Development, and documents perceptions of its impact on the health labour market against the backdrop of the costs of implementation. Through interviews with end users and implementers in six different settings, we document pre-implementation data challenges and consider how the HRHIS has been perceived to affect human resources decision-making and the healthcare employment environment.

**Discussion and evaluation:**

This multisite case study documented a range of perceived benefits of Uganda’s HRHIS through interviews with end users that sought to capture the baseline (or pre-implementation) state of affairs, the perceived impact of the HRHIS and the monetary value associated with each benefit. In general, the system appears to be strengthening both demand for health workers (through improved awareness of staffing patterns) and supply (by improving licensing, recruitment and competency of the health workforce). This heightened ability to identify high-value employees makes the health sector more competitive for high-quality workers, and this elevation of the health workforce also has broader implications for health system performance and population health.

**Conclusions:**

Overall, it is clear that HRHIS end users in Uganda perceived the system to have significantly improved day-to-day operations as well as longer term institutional mandates. A more efficient and responsive approach to HRH allows the health sector to recruit the best candidates, train employees in needed skills and deploy trained personnel to facilities where there is real demand. This cascade of benefits can extend the impact and rewards of working in the health sector, which elevates the health system as a whole.

## Background

Human resources for health (HRH) are a linchpin in the delivery of healthcare but can also represent a barrier in low-resource settings where human resources are in short supply and/or poorly managed [1]. Successful human resources management is not only resource-intensive but also information-intensive [2–4]. Each stage of the HRH life cycle is associated with myriad data that must be maintained and checked to ensure a sufficient and efficient health workforce [4]. Policymaking requires aggregating data from individual health facilities to the regional, district and/or national levels. Without timely and complete information, human resources cannot be allocated effectively [2]. Thus, information shortages can compound resource shortages.

To address the need for timely and comprehensive HRH information, governments and organizations have been actively investing in electronic health information interventions, including in low-resource settings [3]. These investments are grounded in the belief that electronic approaches to data management improve the capture, portability and use of data for decision-making. The theoretically plausible value proposition underpinning electronic human resources information systems (HRISs) is that such systems strengthen management of HRH data [5], which in turn has the potential to create a more efficient and transparent work environment. This additional transparency and responsiveness can improve job opportunities within the health sector, resulting in a more effective health workforce that should in turn elevate the delivery of health services [1, 6]. The economics of HRIS in low-resource settings are not well understood, however, and warrant investigation and validation.

In Uganda, HRH are severely constrained, with 1 health professional per 700 people [7]. The World Health Organization (WHO) defines countries as “HRH crisis countries” if they have less than 1 health professional per 435 people (2.3 per 1000) [2]. According to this definition, Uganda is close to two times beyond the HRH crisis threshold. To improve its HRH infrastructure and alleviate the HRH crisis, Uganda began developing a national HRIS in 2005, called the Human Resources for Health Information System (HRHIS). To implement the HRHIS, Uganda adapted the iHRIS open source software (www.ihris.org) developed through the global United States Agency for International Development (USAID)-funded Capacity and Capacity*Plus* projects led by IntraHealth International.

The long-term sustainability and expansion of any HRIS requires comparing the reality of a given HRIS with its theoretical strengths, and, more specifically, evaluating any benefits realized against the costs incurred. This case study describes the implementation of Uganda’s HRHIS and evaluates its perceived impact on the health labour market against the backdrop of the costs of implementation. Through interviews with end users and implementers in six different settings, we document pre-implementation data challenges and consider how the HRHIS is perceived to have affected human resources decision-making and the healthcare employment environment.

## Case description

### Adoption decision

In 2006, the USAID-funded Capacity Project began working with health workforce stakeholders in Uganda to address their information challenges. The Capacity Project worked with country stakeholders to document Uganda’s requirements and develop and test the iHRIS software, which in 2006 was adopted for the national HRHIS. USAID provided ongoing technical assistance for adapting and rolling out HRHIS through the global projects and the bilateral Uganda Capacity Program.

### iHRIS components

iHRIS is divided into five distinct applications to meet different health workforce stakeholder needs, including iHRIS Manage, iHRIS Qualify, iHRIS Train, iHRIS Plan and iHRIS Retain [8]. In the case of the Uganda HRHIS, these different applications work together as parts of a larger interoperable national health workforce information system. First, Uganda uses country-specific builds of the iHRIS Manage software in 81 districts, 14 regional referral hospitals and 2 national referral hospitals. Second, the four national health professional councils use the iHRIS Qualify software to register and license 52 231 doctors, nurses, midwives, pharmacists and allied health professionals.

## Methods

This study examined the impact of the Uganda HRHIS by documenting the perceived benefits and costs of the system at six different sites. These sites engage with the HRHIS in a variety of ways and perform a variety of human resources management functions. At each site, a semi-structured interview was conducted with one end user to capture perceptions about the benefits of the HRHIS, and system costs were gathered through interviews with the Uganda Capacity Program’s HRIS implementation team members. Retrospective informed consent was obtained from each participant. This study was determined to be exempt by IntraHealth International’s Department of Monitoring, Evaluation, and Research.

The interviews with end users sought to gather feedback about the data-related responsibilities of each institution as well as the pre-implementation data challenges that motivated the specific decision to adopt an HRIS. This was followed by an inquiry about how tasks had changed or improved after the HRHIS was implemented. Questions such as “how was this task performed before iHRIS was in place?”, with a specific request to “include items such as number of people involved, amount of time required, and other resources used” were used to understand the resources required. The change in process and resources was then approached through questions such as “In your opinion, how has iHRIS changed this task? How has it changed the steps associated with this task? How has it changed the resources (personnel hours, physical resources) required for this task?”

Capturing benefits was intentionally approached in a relatively unstructured way due to the breadth of the HRHIS and the desire to elicit value as defined by actual users of the system. Thus, rather than rating a series of pre-established benefit categories, the interviews drew out perceived benefits through open-ended discussions. For each type of benefit identified by end users, the goal of the interviews was to identify how a given task was performed before implementation, understand how the HRHIS changed the way the task was conducted, draw inferences about the benefits and impact of the system and assign a monetary value to each benefit using an ingredient-based approach [9]. The monetary value was calculated by documenting the change in resources involved post-implementation and assigning monetary values to those resources (employee time, printing supplies, etc.). All monetary values are given in 2014 U.S. dollars ($). As this is a static analysis, no discount rate is applied.

The interviews with implementation team members were more highly structured and focused on identifying implementation components and their associated costs. Implementation costs are organized into five categories: hardware, software installation and network costs, training, data entry and cleaning and follow-up technical support. Within each category, the major components were identified. For example, within the hardware category, items such as servers, power supply, power backup and computers were identified separately. Each of these items was described individually, and a unit cost and number of units were ascribed to each. Questions about servers, for example, included questions about cost as well as other hardware included or associated with the server, such as laptops and network electronics. In terms of technical support, participants were asked how many days were required for HRHIS technical follow-up, the type of individuals involved and their daily compensation.

During the interview, notes were recorded on templates designed for each type of interview (benefits and costs). They were then individually summarized in reports that also quantified the resource savings associated with the HRHIS.

### Sites

Table 1 summarizes the six HRHIS case study sites. The six institutions reflect a variety of healthcare system roles – including service delivery, workforce regulation, workforce training, staffing oversight and policymaking – providing a diverse representation of HRH management responsibilities and offering a broad depiction of how organizations in Uganda use and benefit from the HRHIS.

**Table 1:** Description of case study sites

Site	iHRIS application(s)	Charge	Primary data-related functions
Allied Health Professionals Council (AHPC)	Qualify	Regulate and control practice, education and training of allied health professionals through registration, licensing, supervision and monitoring	Registration of new graduates, annual licence renewals, registration and inspection of facilities and clinics
Health Tutors College (HTC)	Train	Offer postgraduate training to candidates who already hold diplomas or degrees in one of the approved nurses, midwifery and allied related specialties, with the goal of creating well-informed and effective tutors	Tracking of progress on curriculum implementation and student performance; collection of information on faculty, tutors and training experience of individual health workers
Ministry of Health Human Resources Development (HRD)	Train	Manage all matters related to HRH policy and strategic framework that relate to training based on the training needs assessment and planning of the health workforce	Coordination of in-service training
Ministry of Health Human Resources Management (HRM)	Manage	Identify and manage HRH needs; project HRH supply	Determination of staffing levels and recruitment planning, performance management and appraisals, promotions and retirement management and succession of the country’s healthcare workforce
Uganda Nurses and Midwives Examination Board (UNMEB)	Train	Streamline, regulate and coordinate examinations and awards for nurses and midwives in Uganda	Management of practical exams; generation of examination cards, student albums, individual certificates and results record books; generation of examination data
Uganda Protestant Medical Bureau (UPMB)	Manage	Support Protestant churches involved in provision of health services	Management, planning and development of UPMB health workforce

The facilities chosen represented a range of other characteristics that are relevant to the cost and value of health workforce information systems. For example, the location of a facility has implications for the costs associated with training and providing ongoing technical support. Other efficiencies are also achievable when institutions are co-located, as is the case with the Ministry of Health’s Human Resources Management and Human Resources Development offices, which are two of the sites explored here.

### Benefits

Respondents identified three main HRHIS functions when discussing perceived benefits associated with the information system: registration and licensing, staffing, and training.

#### Registration and licensing

Two of the sites included in this study, the Allied Health Professionals Council (AHPC) and the Uganda Nurses and Midwives Examination Board (UNMEB), have responsibilities pertaining to registration and licensing. AHPC directly oversees registration and licence renewal for personnel as well as facilities and clinics. The UNMEB annually administers a promotion exam to all nurses and midwives in training as well as the final summative professional exam for nurses and midwives. Users at both sites stated that information was not systematically maintained before the HRHIS was implemented. For AHPC, this resulted in delays in licence renewals, and UNMEB cited issues with exam integrity, both of which threaten the ability to ensure a qualified health workforce.

At AHPC, the annual process of licence renewal was particularly challenging before the HRHIS was installed because the registration clerk had to locate the registrant’s file among approximately 5000 records that were not filed in a systematic manner. According to the users interviewed, the process for a single renewal took 4–8 h and occasionally as long as a week. Using the iHRIS software dramatically reduced the time involved in these renewals; the process now takes approximately 10 min per renewal. The value of this time savings is shown in Table 2.

**Table 2:** Value of efficiency gains for Allied Health Professionals Council (AHPC) licence renewals (for 100 renewals)

Function	Unit cost	Impact	Total cost
Registrant’s record retrieval	$0.91/h	4 h and 50 min per record	$439.83
Total			$439.83

Users at UNMEB specifically spoke about improved exam integrity after the HRHIS was implemented. The use of iHRIS Train software helps to ensure that students are taking their own exams and are assigned the correct result. Prior to the HRHIS, students would occasionally try to claim the results of those with similar names or would claim that they took the exam but did not receive results. Users stated that the HRHIS reduced the frequency of these problems.

Other examples of benefits associated with the HRHIS related to the timeliness of records. Both AHPC and UNMEB described more accurate and timely records using the system. In terms of improving the data used for decision- and policymaking, users stated that better data could also be more easily compiled into reports for stakeholders and policymakers and that the HRHIS had facilitated improved information-sharing with entities such as the Ministry of Health and professional councils.

By improving the efficiency of licence renewals and the integrity of licensing exams, the HRHIS was perceived to have improved the employment environment. Faster licence renewals reduce interruptions in care delivery, while improved exam integrity operates even earlier in the human resources life cycle by ensuring that only qualified individuals are allowed to practise. Both of these HRH-strengthening effects are likely to make the health sector more attractive to high-quality workers by rewarding performance and reducing administrative distractions.

#### Staffing

Adequate staffing of health facilities requires an understanding of supply and demand, specifically how to allocate the health workforce to meet the various healthcare needs of the population. The inevitable geographic variation in health status and disease burden also speaks to the distributional challenges of managing staffing. The Ministry of Health’s Human Resources Management (HRM) office manages the health workforce at the national level and is also tasked with projecting the future supply of health workers. The Ugandan Protestant Medical Bureau (UPMB) works within a narrower scope, supporting the HRH needs of the 278 healthcare facilities associated with Ugandan Protestant churches. Both currently use the iHRIS Manage software component to address staffing and aggregate data from their partner facilities.

Prior to implementing the HRHIS, HRM lacked access to accurate data to enable evidence-based decisions regarding staffing for the country’s health workforce. The data to support these decisions existed but was fragmented, held in different offices on personal computers and stored in different formats, inhibiting the comprehensive analysis at the core of HRM’s charge. To produce a comprehensive annual report for HRM on workforce exit and attrition, it took two people 2 months to collect and collate the data and present them in the format requested by the ministry. Requests for information, sent by mail to the districts, were met with a high rate of non-response, resulting in reports that were incomplete. After implementation of the HRHIS, the HRM human resources manager obtained access to accurate and comprehensive data on all health employees working in public health facilities and paid by the ministry. It now takes the manager less than 15 min to generate a report on workforce exit and attrition. Savings are also realized due to the electronic entry of data, which reduces costs associated with tasks such as photocopying. The monetary value of this improved efficiency in HRM report generation is shown in Table 3.

**Table 3:** Value of efficiency gains for Ministry of Health’s Human Resources Management (HRM) report generation

Function	Unit cost	Impact	Total cost
Report preparation	$8.73/day	79.97 days	$697.82
Other costs (photocopying, printing, telephone calls, postage, incidentals)	$698.08	1	$698.08
Total			$1395.90

Another benefit directly attributed to HRM’s use of iHRIS Manage was the nationwide mass recruitment of health workers. The HRHIS allowed HRM to identify gaps in staffing, which were presented to the Ugandan Parliament and resulted in government funding for the recruitment of 6172 additional health workers as well as salary enhancement for some doctors. Other benefits cited by respondents at HRM included an increased percentage of approved posts filled by trained health workers, improved recruitment and retention of health staff and improved allocation of workers to underserved areas.

On a smaller scale, UPMB faced similar challenges in HRH management, despite recognizing that workforce strengthening was one way to improve its organizational effectiveness. Previously, there was no centralized database where all the employee records were held, and manual reports from each facility were often incomplete, inaccurate or significantly delayed. The lack of oversight made it difficult to verify that individuals being paid had actually been officially hired and were reporting for work. UPMB now uses iHRIS Manage (as a separate system from the Uganda public sector HRHIS) to monitor staffing in 25 of its facilities comprising records on over 4000 health workers. End users stated that the iHRIS software has improved the accuracy and efficiency of compiling monthly staff returns through a heightened ability to track health workers. This tracking was stated as facilitating retention because UPMB can now identify employees who qualify for salary increases, scholarship programmes and training. This information has also improved sharing of data with the Ministry of Health and other stakeholders.

At HRM and UPMB, the iHRIS software has empowered stakeholders and policymakers to make informed staffing decisions. This improves the supply of health workers because the health workforce is allocated more efficiently and fairly. Not only can workers be allocated to areas of greatest need where they can have the most impact, but they can be more quickly identified for transfers, promotions, salary increases and other perks. These supply-side improvements create a more transparent and rewarding work environment, which can be used to attract and retain high-calibre health workers. Ultimately, staffing improvements can translate to health service delivery improvements resulting from the improved ability to meet staffing needs and retain high-quality workers.

#### Training

The Ministry of Health Human Resources Development (HRD) department and the Health Tutors College (HTC) are both charged with advancing the training of health workers to meet current and future population health needs but at different scales. The HRD coordinates in-service training at the national level. The HTC’s more limited mission is to improve the skills of health tutors (those who train health workers). Both institutions currently use the iHRIS Train component of the iHRIS software to support their activities.

Before the implementation of iHRIS Train, HRD’s coordination of in-service training was described as non-existent. The whole process was characterized by incomplete data, making it difficult to determine who was trained, by whom, when, where and on what topics. Training partners held this information in their systems since there were no formal processes to exchange these data. Despite the challenges, the HRD was charged with generating annual in-service training reports. This task took 2 months for two people working full-time. The process involved requesting data from trainers in the districts and training institutions by e-mail, phone and/or postal mail. Trainers or institutions often did not respond or sent incomplete information about participants, trainers or training content. As a result of iHRIS Train implementation, generation of a report now takes the HRD less than 10 min, with the added feature of being able to sort the data by cadre, facility or health district. In addition, there are also reductions in the cost of materials used in requesting data, such as printing and postage. The value of the resources saved on report generation using the HRHIS are summarized in Table 4.

**Table 4:** Value of efficiency gains for Ministry of Health Human Resources Development (HRD) annual report generation

Function	Unit cost	Impact	Total cost
Report preparation	$8.73/day	39.5 days	$344.68
Other costs (photocopying, printing, telephone calls, postage, incidentals)	$698.08	1	$698.08
Total			$1042.76

The HRHIS allows training coordinators to identify and plan for needed training, coordinate training attendance with supervisors, track who has been trained and award continuous professional development credits. Using iHRIS Train, the system also provides information that is being used to select courses and trainees, and it improves the alignment of courses with identified needs. Overall, it empowers HRD in terms of controlling who registers for trainings, reducing duplicate courses and allocating training resources more efficiently.

At the HTC, the iHRIS Train software component of the HRHIS has enabled the college to track progress on curriculum implementation and student performance and collect information on faculty, tutors and the training experience of individual health workers. Before its implementation, the college management lacked the accurate and accessible data needed to make evidence-based decisions. Previously, students’ registration information was contained in notebooks; no information was maintained on faculty members’ demographic data, training or skills; and there were no systematic records of student fee payments, dropouts, academic progress or disciplinary actions. At present, HTC administrators use the HRHIS to more closely monitor student progress and faculty composition and obtain timely and accurate data on student admissions and dropouts as well as faculty-student ratios and training specializations.

The use of the HRHIS for monitoring training by HRD and HTC allows for easier dissemination and application of up-to-date information within the health workforce. By strategically determining training offerings, the HRD can selectively strengthen the workforce to meet current and future health needs. HTC’s use of the information system, meanwhile, contributes to a more rigorous training environment, which will eventually translate into the production of better teachers. In addition, the HRHIS facilitates the addition of new training programmes as the need arises, improving the overall flexibility of the training infrastructure. A more responsive and tailored training strategy can improve health service delivery by meeting HRH demands for new skills and services.

### Implementation costs

Table 5 summarizes the different categories of costs faced by the six Ugandan sites in their implementation of the HRHIS. While we are not able to provide details about why certain costs differ across sites, this broad comparison does permit identification of those costs that are either relatively uniform or variable across sites. Training costs exhibited the most variation, while costs in areas such as installation, data entry and technical support were fairly consistent across sites.

**Table 5:** Summary of implementation costs

	AHPC	HTC	HRD^a^	HRM^a^	UNMEB	UPMB
Hardware	$977	$1920	$1850		$2199	$1117
Software installation and network costs	$768	$698	$873		$593	$768
Training	$4887	$1326	$1990	$1082	$1501	$2443
Data entry and cleaning	$880	$817	$775	$775	$775	$859
Technical follow-up	$440	$440	$405	$405	$405	$405
Total	$7951	$5201	$4530	$3623	$5473	$5592

^a^HRD and HRM share a server

## Discussion and evaluation

This multisite case study documented a range of perceived benefits of Uganda’s HRHIS through interviews with end users that sought to capture the baseline (or pre-implementation) state of affairs, the perceived impact of the HRHIS and the monetary value associated with each benefit. In general, the system appears to be strengthening both demand for health workers (through improved awareness of staffing patterns) and supply (by improving licensing, recruitment and retention of the health workforce). The dire state of pre-implementation data management in most of the institutions allowed for the investment in a HRIS to generate substantial gains by improving the speed and accuracy of existing tasks and supporting additional tasks and activities. For example, report generation was a time-intensive activity pre-HRHIS, requiring months of work by multiple employees; with the HRHIS, reports are generated in a matter of minutes. Moreover, by supplying accurate information about the distribution, qualifications and employment records of health workers, the HRHIS makes it possible to implement new initiatives such as offering enhanced retention incentives.

This case study offers evidence that an HRHIS can be used to galvanize efficiency and equity improvements in the health workforce. There is a wealth of evidence suggesting that health workers are discouraged by inadequate HR management and being underutilized [10–13]. Improved transparency empowers the health sector to identify high-quality workers at every stage of the HRH life cycle, information that can be used to recruit, retain and reward these workers. This heightened ability to identify high-value employees makes the health sector more competitive for high-quality workers, and this elevation of the health workforce also has broader implications for health system performance and population health. Chen et al. [14] defines an HRH policy framework comprised of three objectives: coverage, motivation and competence. Based on this framework, interventions that strengthen these attributes result in population health improvements through more equitable access, improved efficiency and effectiveness and heightened quality and responsiveness [6].

This study focused not only on describing perceived benefits of the HRHIS but also on contextualizing them in terms of the costs of implementation. This dual focus on benefits and costs can be enlightening because, while the costs of implementation and operation of an HRIS are fairly tangibly realized, the benefits tend to be somewhat more elusive [15, 16]. A server can be directly paid for and seen, for example, whereas the ability to locate records more efficiently has an incremental impact that lacks an immediately observable monetary value. By juxtaposing costs and benefits, this case study underscores the magnitude of benefits relative to the costs of implementation.

In considering the benefits of Uganda’s HRHIS or other HRISs, it is important to note that each of the perceived benefits identified is a recurring benefit. The efficiency gains of report generation, for example, will be realized every time a report is created, and thus, this benefit will accrue perpetually. Moreover, our quantification of benefits here is incomplete; in most cases, it is limited to the labour cost savings. Nonetheless, even with this limitation, the magnitude of benefits ranged from over $400 to just under $1400. At the lower end of the spectrum, the single realized benefit is approximately equivalent to the costs of technical support. At the higher end, the annual realized benefit more than offsets the cost of training or hardware at some sites. Again, it is important to recognize that we are only considering a single source of benefit for each institution, suggesting that it is very likely that the benefits of the HRHIS more than offset the costs of both implementation and operation.

One drawback to a site-specific approach to documenting benefits is that higher level benefits are not captured. For example, the downstream health benefits of hiring and training additional health workers are not realized by any one institution, despite being a significant benefit and representing the overarching mission of all of the organizations. In addition, higher level benefits such as improved decision-making due to higher quality and more available data were not captured in this study. Instead, our site-specific approach generated more administrative benefits that accrue to each site individually and thus may underestimate the true value of a stronger national HRHIS. It is also possible that these benefits could be subject to reporting bias or errors. However, we believe this approach was advantageous because these individuals are likely to have a more informed, detailed perspective of the role of the HRHIS and how it has changed HRH management.

The case study also incompletely captured system costs, solely looking at implementation costs and not gathering data on operating and maintenance expenses. The latter include expenses such as electricity, ongoing technical support and updating and additional trainings. These expenses are typically small in magnitude compared to implementation costs. An expanded version of the evaluation framework used in this study should include operating and maintenance costs.

The natural next step is to examine and quantify a broader array of HRIS benefits in a wider range of facilities and countries, including the higher level policymaking and labour market efficiency benefits described previously. In particular, this case study captured perceived benefits, so further work can provide evidence to substantiate and more precisely document these improvements. For example, while it is known that HRHIS contributed to the recruitment of additional health workers and salary enhancements for some physicians, further data could be gathered regarding the impact of HRHIS on the vacancy rate and remuneration. In addition, there are other aspects of the health system that we could examine, such as pre-service training. Given that the iHRIS software has been adopted widely in low-resource settings to aid in HRH data management, opportunities abound to extend this work [17]. Even without further studies, however, the consistency across interviews and sites in Uganda regarding HRHIS benefits suggests that the on-the-ground implementation realities reflect the theory behind HRISs, namely that such systems can dramatically improve the capture and use of data for health workforce decision-making.

Any future work to examine a broader scope of benefits, facilities and countries should also attempt to identify efficiencies and improvements that can be made on the way that the iHRIS software is implemented and used at different sites. Assessing variations in implementation and use can be the basis for identifying ways to improve the return on investment in the system.

## Conclusions

Overall, it is clear that HRHIS end users in Uganda perceived the system to have significantly improved day-to-day operations as well as longer term institutional mandates. The six organizations previously were hampered by the lack of correct, complete and timely data, a shortcoming that in some cases completely undermined their organizational mission – such as HRD’s management of in-service training. The benefits described in the Uganda case study were largely efficiency gains for existing tasks; this type of benefit is incremental in nature but also recurs indefinitely. On the other hand, because many of the HR processes were extremely resource-intensive (for example, time and labour) prior to the HRHIS, the efficiency gains translate into significant resource savings after implementation. In addition to further identifying the benefits of the HRHIS, future work should tackle the next step in the chain of effects and understand how the intervention has indirectly contributed to improved health outcomes. Given the limited resources and multitude of health sector demands in settings such as Uganda, developing an evidence base around both the costs and benefits of HRIS is important for promoting broad adoption of HRIS investments as well as the sustainability of existing implementations.

The immediate effect of an HRIS is improved data management, which has the potential to translate into more informed decision-making and more tailored policies and ultimately galvanize improvements in the broader health system [14]. A more efficient and responsive approach to HRH allows the health sector to recruit the best candidates, train employees in needed skills and deploy trained personnel to facilities where there is real demand. This cascade of benefits can extend the impact and rewards of working in the health sector, which elevates the health system as a whole. More committed and knowledgeable health workers – located in the right facilities and equipped with the right skills – will result in better health service coverage and, ultimately, better population health outcomes. Although the full cost of implementation of systems such as the HRHIS can sometimes appear daunting, our findings suggest that the benefits of HRIS investments are similarly staggering in magnitude and have the advantage of being long-lasting.

## Abbreviations

AHPC: Allied Health Professionals Council
HRD: Ministry of Health Human Resources Development
HRH: Human resources for health
HRHIS: Human Resources for Health Information System (as installed to support the Uganda public health sector)
HRIS: Human resources information system
HRM: Ministry of Health Human Resources Management
HTC: Health Tutors College
UNMEB: Uganda Nurses and Midwives Examination Board
UPMB: Uganda Protestant Medical Bureau
USAID: United States Agency for International Development
WHO: World Health Organization
